# 3β, 6β-dichloro-5-hydroxy-5α-cholestane facilitates neuronal development through modulating TrkA signaling regulated proteins in primary hippocampal neuron

**DOI:** 10.1038/s41598-019-55364-8

**Published:** 2019-12-12

**Authors:** Md. Abdul Hannan, Md. Nazmul Haque, Raju Dash, Mahboob Alam, Il Soo Moon

**Affiliations:** 1 Department of Anatomy, Dongguk University College of Medicine, Gyeongju, 38066 Republic of Korea; 2 Department of Biochemistry and Molecular Biology, Bangladesh Agricultural University, Mymensingh, 2202 Bangladesh; 3 Division of Chemistry and Biotechnology, Dongguk University, Gyeongju, 780-714 Republic of Korea; 4 Dept. of Fisheries Biology and Genetics Patuakhali Science and Technology University, Patuakhali, 8602 Bangladesh

**Keywords:** Neurotrophic factors, Cellular neuroscience

## Abstract

Potentiating neuritogenesis through pharmacological intervention might hold therapeutic promise in neurodegenerative disorders and acute brain injury. Here, we investigated the novel neuritogenic potentials of a steroidal chlorohydrin, 3β, 6β-dichloro-5-hydroxy-5α-cholestane (hereafter, SCH) and the change in cellular proteome to gain insight into the underlying mechanism of its neurotrophic activity in hippocampal neurons. Morphometric analysis showed that SCH promoted early neuronal differentiation, dendritic arborization and axonal maturation. Proteomic and bioinformatic analysis revealed that SCH induced upregulation of several proteins, including those associated with neuronal differentiation and development. Immunocytochemical data further indicates that SCH-treated neurons showed upregulation of Hnrnpa2b1 and Map1b, validating their proteomic profiles. In addition, a protein-protein interaction network analysis identified TrkA as a potential target connecting most of the upregulated proteins. The neurite outgrowth effect of SCH was suppressed by TrkA inhibitor, GW441756, verifying TrkA-dependent activity of SCH, which further supports the connection of TrkA with the upregulated proteins. Also, the computational analysis revealed that SCH interacts with the NGF-binding domain of TrkA through Phe327 and Asn355. Collectively, our findings provide evidence that SCH promotes neuronal development via upregulating TrkA-signaling proteins and suggest that SCH could be a promising therapeutic agent in the prevention and treatment of neurodegenerative disorders.

## Introduction

Neurodegenerative disorders are characterized by the substantial loss of neurites as well as whole neurons, due to the insufficient trophic support from various growth factors^1,2^. Acute brain injury such as stroke or trauma also results in extensive damage to neuronal circuitry. Neurotrophic factors (NTFs) have been shown to promote neuronal maturation as well as survival^3–5^. In experimental animal models, neurological disorders and stroke are successfully treated with the agents that modulate NTF receptor activity^6^. Therefore, NTF mimetics with the ability to reconstruct damaged neuronal circuitry through modulating neuritogenesis in an NTF-dependent or independent manner might hold the therapeutic promise in the treatment of neurodegenerative disorders and acute brain injury.

Identification of NTF mimetics may provide significant drug leads to shift the balance from neurodegeneration to neuroregeneration in the various forms of neuronal injuries. In a series of previous studies, we reported several marine and terrestrial origin-natural products that show neurotrophic as well as neuroprotective activities in primary hippocampal culture^7–16^. Moreover, many small molecules, including gambogic^17^, amitriptyline^18^, deoxygedunin^19^, 7,8-dihydroxyflavone^20^, LM22A-4^21^, and withanolide A^22^ have been reported to possess neurotrophic and neuroprotective activities. Also, natural sterol^23–25^ and their derivatives^26,27^ are shown to have neurotrophic and neuroprotective activities. Sterol derivatives are promising drug candidates due to their natural sterol-like pharmacokinetic attributes, including blood-brain barrier permeability and oral bioavailability. Although many efforts have been paid to explore neurotrophin/its mimetics, still there is a lack of potential therapeutic agent in the market. Therefore, our effort was dedicated to exploring potent and druggable pharmacological leads from the natural as well as synthetic sources, which have neuritogenic potentials.

Profiling of total cellular proteome aiming to map global protein expression offers a set of differentially expressed proteins as a response of the cell to pharmacological intervention. This approach successfully elucidates the molecular mechanism underlying the pharmacological action of a drug or chemical substance. In our previous studies, we used a proteomic approach to unravel the underlying molecular mechanism of the neurotrophic activity of a natural substance^12^. Here, following the same approach, we employed proteomic analysis to gain insight into the underlying molecular mechanism of neurotrophic activity of SCH in primary hippocampal neurons. First, we report that SCH significantly enhanced neuronal polarization and axodendritic maturation. Next, we demonstrate that neurons treated with SCH showed upregulation of multiple proteins correlated with their development and maturation. Bioinformatics analysis identified the interaction of TrkA with most of the altered proteins. Finally, we confirmed that the neuritogenic activity of SCH was dependent on TrkA activation.

## Results

### SCH induces neurite outgrowth in a concentration-dependent manner

**Figure 1:**
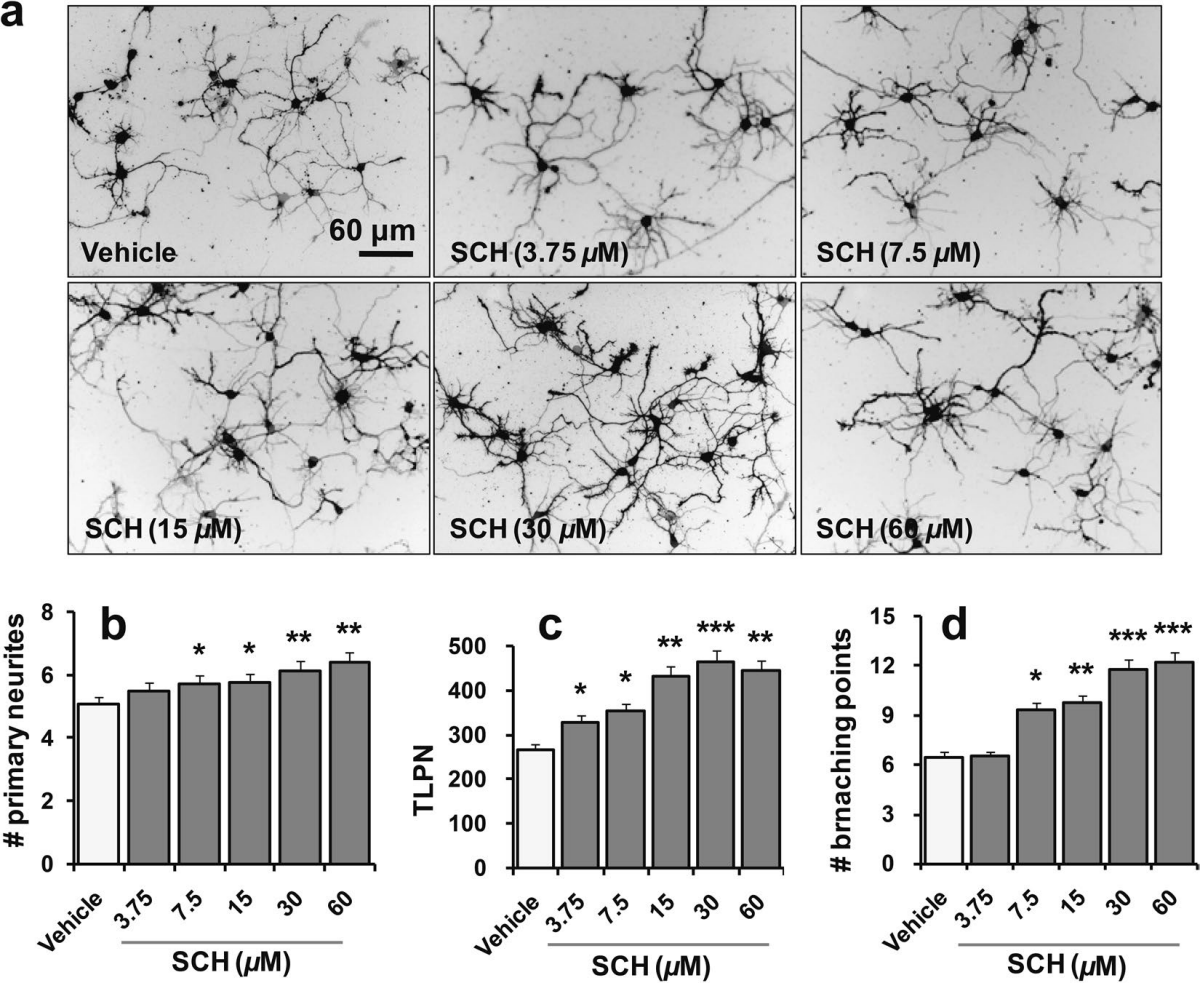
Optimization of the effective concentration of SCH for neurite outgrowth activity. Primary culture of embryonic hippocampal neurons was incubated with vehicle [DMSO, <0.5% (v/v)] or SCH at indicated concentrations for three days. Representative photomicrographs from the cultures incubated with different concentrations of SCH (**a**). Dio-stained images were inverted and presented as a grayscale mode with black neurite path and white background. Scale bar, 60 μm, applies to all images. Morphometric analysis for the number (**b**) and the total length of primary neurites, TLPN (**c**), and branching points (**d**) demonstrates that SCH promotes neurite outgrowth in a concentration-dependent manner. Statistical significance compared to vehicle: *p < 0.05, **p < 0.01 and ***p < 0.001 (ANOVA). Data points represent the mean ± SEM (n = 20 individual neurons). SEM, standard error of mean.

To establish the compound’s effective concentration, we cultured hippocampal neurons in a medium treated with SCH ranging from 3.75  to 60 μM concentrations for three days. SCH substantially increased the number and the length of primary processes and their branches as well in a concentration-dependent manner (Fig. 1). As 30 μM concentration exhibited the highest neurite outgrowth promoting activity without showing any toxic effect, we used SCH at this concentration for all of the subsequent studies unless stated otherwise. In addition, to establish the maximum subtoxic dose, we exposed neurons to a varying concentration of SCH for the subsequent seven days. Cell viability and cytotoxicity studies using trypan blue exclusion and LDH activity assays, respectively, revealed that doses up to 120 μM were nontoxic to cultured neurons, instead significantly promoted their survival (*p* < 0.05) (Supplementary Fig. S1).

### SCH modulates neuronal polarity

**Figure 2:**
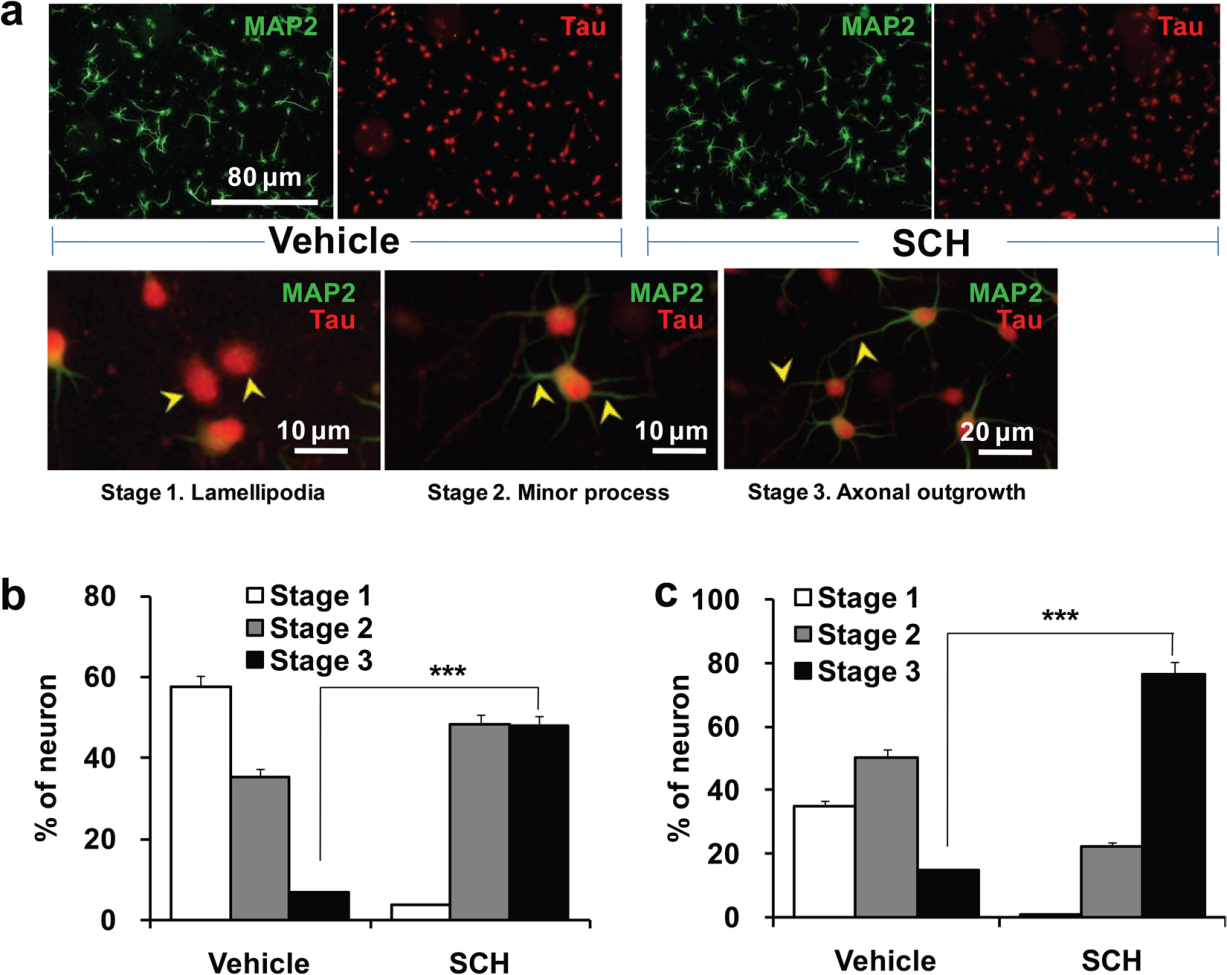
Effects of SCH on the neuronal polarity. Neurons were grown on the same culture conditions as indicated in Fig. 1 for 24 hrs and 48 hrs, and then fixed and double-immunostained for MAP2 (green) and Tau (red), an axon and a dendrite specific markers, respectively. Fluorescent images showing early developmental stages (**a**); Lower panel: stage 1 (lamellipodia stage, indicated by arrow-heads), stage 2 (minor process stage, indicated by arrow-heads), stage 3 (axonal sprouting, indicated by arrow-heads). Scale bar, 80 μm, applies to all images of the upper panel. Percentage of neurons that reached different developmental stages at 24 hr (**b**) and 48 hr (**c**) of incubation. Statistical significance compared to vehicle: ***p < 0.001 (Student’s *t*-test). Data points represent the mean ± SEM (n = 3, each replicate with ~1000 neurons). SEM, standard error of the mean.

Morphological polarity of a multipolar neuron is determined by the specialization of neuronal processes into axon and dendrites. Such polarization into domains specialized for either receiving (dendrites) or transmitting (axons) neuronal signals forms the basis for all neural circuitry^28^. It is, therefore, worth evaluating whether SCH could function as a catalyst for the early neurodevelopmental events during morphological polarization of neurons. To this goal, we categorized neurons into different developmental stages^29^ based on their morphological features and immunocytochemical indication. We used specific markers to axon (anti-Tau antibody labeled) and dendrites (anti-MAP2 antibody labeled) to differentiate the developmental stages. At this transition stage of polarization, we observed some neurons with anti-MAP2 antibody labeled processes, while axons were already differentiated by anti-Tau antibody. We categorized those neurons under stage III. However, a substantial variation in the number of neurons in each of the developmental stages was observed in SCH-treated cultures when compared to control ones, suggesting an early onset of the neuritogenic activity of SCH (Fig. 2a). Within 24 h, half of the total neurons offered with SCH treatment showed axonal sprouting (stage III), whereas control culture just began to sprout (only 7% of the total cells contained differentiated axon) (Fig. 2b). Within 48 h, 3/4^th^ of the total cells under SCH treatment already reached stage III, whereas 85% of total cells in the control cultures yet to be reached stage III. Moreover, one-third of the total cells in the control cultures still lacked a visible process (stage I), whereas almost every cell in SCH-treated culture already sprouted at least one process (Fig. 2c). However, compared to the previous report^29^, neurons in our culture slowly differentiated to become polarized, for example 93% of the total neurons failed to reach stage III within a day after culture, because we maintained our culture in a compromised conditioning (using serum-free supplement) that allowed the neurons slowly differentiated without hampering cell survival, which eventually offered an ideal assay system for the analysis of neuromodulatory activity of a neuroactive substance^8^. Interestingly, SCH was so efficient in modulating neuronal development that its effect on the early neurodevelopmental events was highly significant (*p* < 0.001) over the vehicle counterpart from the very early stage.

### SCH enhances dendritic arborization

**Figure 3:**
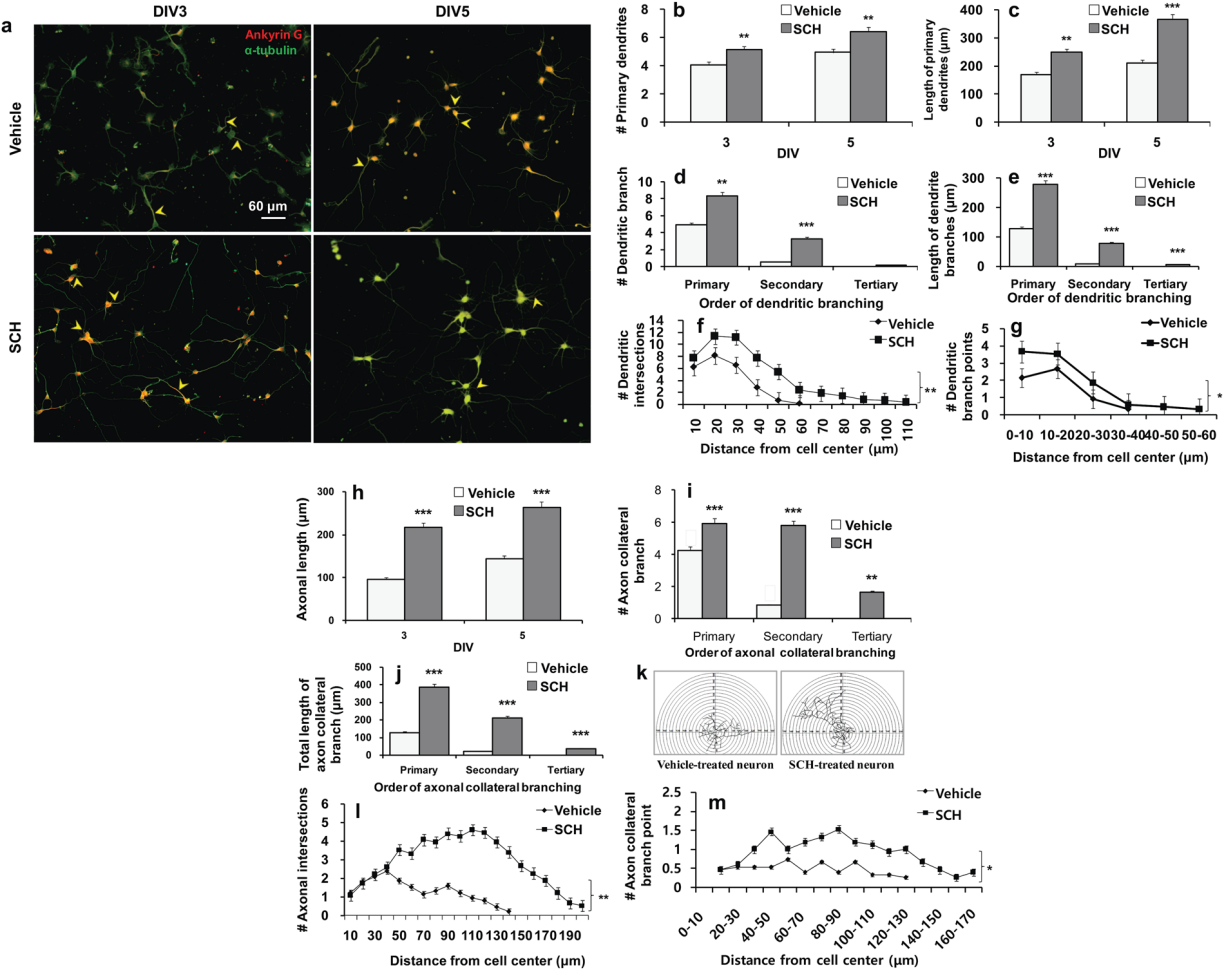
Effects of SCH on axodendritic morphogenesis in hippocampal neurons. Neurons were grown on the same culture conditions as indicated in Fig. 1 for 3–5 days. Neurons were then fixed and double-immunostained for ankyrin G (red) and α-tubulin (green). Ankyrin G is characteristically concentrated at axon initial segment (AIS; indicated by arrows). Representative fluorescent images from cultures at DIV3 or 5 to visualize the cell morphology. Scale bar, 60 μm, applies to all images (**a**). Morphometric analysis of DIV3~5 neurons for dendritic development: the number of primary dendrites (**b**), the length of primary dendrites (**c**), the number of dendritic branches (**d**) and the length of dendritic branches (**e**). Sholl analysis for dendritic intersections (**f**) and branch points (**g**) in DIV5 neurons. Morphometric analysis of axonal development: length of axonal shaft (**h**), axonal collateral branch (**i**) and the total length of axonal collateral branch (**j**) in orders. Sholl image with reconstructed neurons, which does not represent any of the experimental data (**k**). Sholl analysis for axonal intersections (**l**), and axonal collateral branch points (**m**). Bars represent the mean ± SEM (n  >15 neurons). Data points represent the mean ± SEM (n = 15 neurons). Statistical significance compared to vehicle: *p < 0.05, **p < 0.01 and ***p < 0.001 (Student’s *t*-test).

Dendritic arborization is a prerequisite for proper neuronal connectivity. We next, therefore, analyzed whether SCH could affect dendritic arborization at early developmental stages (DIV 3 & 5). Here to note that we performed morphometric analysis until DIV5 because neurons treated with SCH grow so fast that the processes, particularly the axon intermingle with that of the neighboring neurons making the analysis almost impossible after DIV5. It was observed that neurons in SCH-treated culture exhibited a substantial difference in dendritic growth when compared to that of control culture (Fig. 3a). SCH showed a significant increase in the number and total length of primary dendrites (~ 20% and 50–70%, respectively, over control, *p* < 0.01) (Fig. 3b,c). In case of dendritic branching, both the number and length of primary and secondary branches were increased in SCH-treated neurons (Fig. 3d,e). Notably, there were a number of SCH-treated neurons that were even furnished with tertiary branches.

To further characterize dendritic arborization, we employed Sholl analysis that indicated an increase in dendritic complexity in SCH-treated neurons. As shown in Fig. 3f, there was a ~two-fold increase in the number of dendritic intersections in SCH-treated neurons. Whereas the dendritic intersections were extended up to the circle of 110 μm in SCH-treated neurons, they were observed only upto 60 μm in control neurons. Moreover, counting the branching points between two succeeding concentric Sholl’s circles, we observed that there was a significant increase (~1.75-fold, *p* < 0.01) in branching points in SCH-treated neurons (Fig. 3g). In a similar trend with intersections, whereas branching points were extended up to the circle of 60 μm in SCH-treated neurons, there was none beyond 40 μm in control neurons. Together these findings indicate that SCH promoted dendritic development which commenced in the early developmental stage and continued towards the formation of complex arbor.

### SCH facilitates axonal maturation

Given that axonal sprouting and maturation comprise a critical step to establish functional network connectivity, we next evaluated whether SCH could influence axonal development. As illustrated in Fig. 3a, neurons-treated with SCH exhibited an extensive growth of axon over the experimental period. There was a significant increase (~100% over control, *p* < 0.001) in the length of axonal shafts in SCH-treated culture (Fig. 3h). Moreover, SCH significantly increased both the number and length of primary and secondary collateral branches (*p* < 0.001) (Fig. 3i,j). Notably, whereas about a quarter of neurons in SCH-treated cultures developed tertiary branches, none of the control neurons developed the same within the experimental time frame.

Sholl analysis was also carried out to further characterize the axonal maturation. As demonstrated in Fig. 3l, axonal crossings in SCH-treated neurons outnumbered that of control neurons by three to one. Whereas axonal crossings were observed up to the circle of 200 μm in SCH-treated neurons, it was only up to 140 μm in control neurons. In case of collateral branching points, SCH increased the same by 2.5-folds when compared to vehicle (Fig. 3m). Whereas collateral branching points were extended up to 170 μm Sholl circle in SCH-treated neurons, there was none beyond 130 μm in control neurons. Together, these findings indicate that SCH promoted axonal maturation in a similar pattern as in dendritic arborization.

### Proteomic profiles of hippocampal neurons

**Figure 4:**
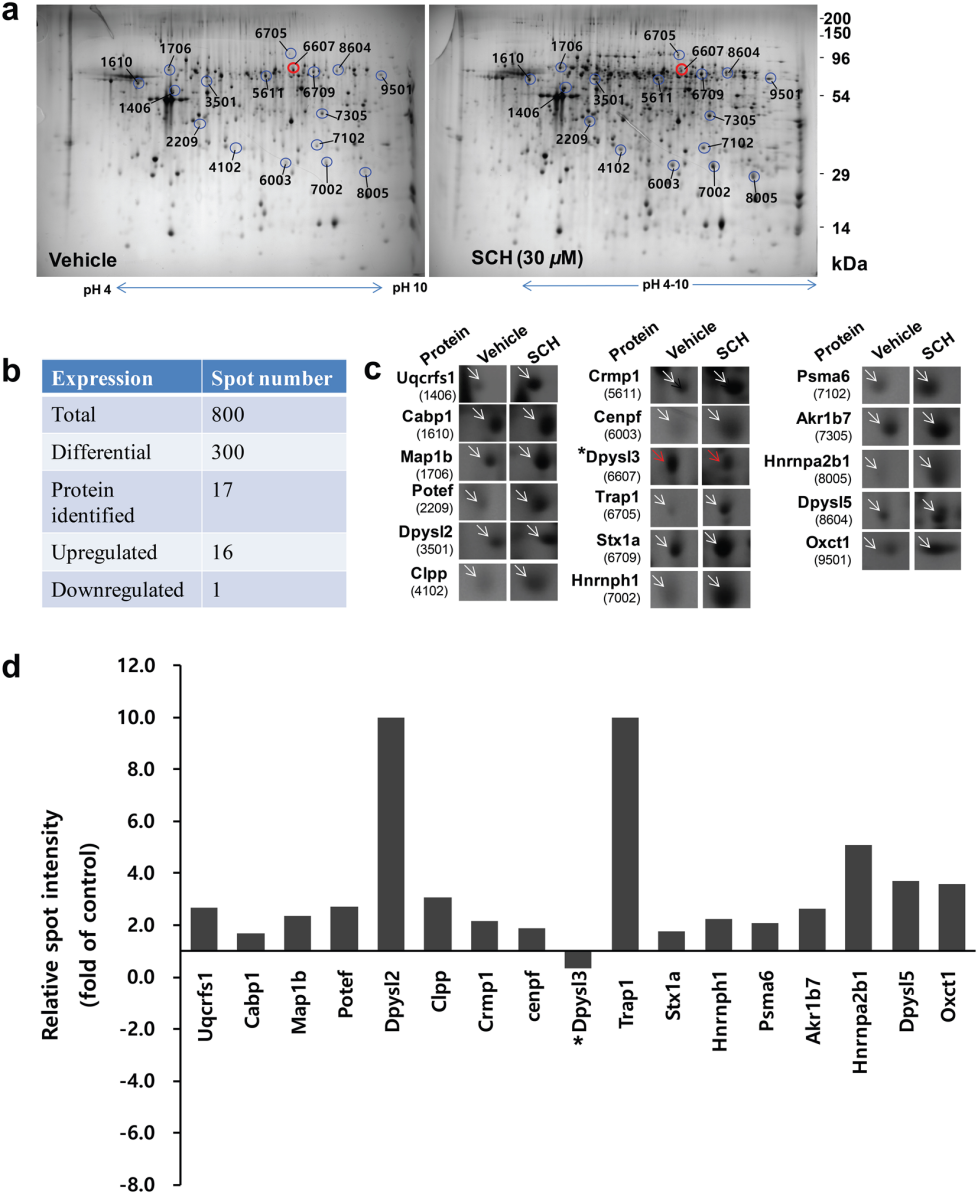
Proteome changes in SCH-treated neurons. Hippocampal neurons were cultured in the presence of vehicle or SCH (30 μM) for 6 days. Proteins were harvested and identified as described in the Supplementary Methods section. (**a**) Representative 2-DE images showing differentially expressed protein spots indicated by circles and labeled with numbers, which correspond to those illustrated in Table 1. Red circles indicate downregulated proteins. The pI range is below the gels, and the molecular mass is on the right of the gels. Summary of the protein spots (**b**). Magnified images of differentially expressed protein spots (**c**). Intensities of differentially expressed proteins (**d**). Relative spot intensities were calculated as ratios versus vehicle-treated controls. Asterisks indicate downregulated spots.

To understand molecular events during early neuronal development, we analyzed cellular proteome in primary hippocampal culture in the presence or absence of SCH treatment using MALDI-TOF-MS-based quantitative proteomic approach. In primary neuronal cultures, the neurite extension activity of SCH commenced as soon as undifferentiated neurons attached to the surface. Although dendritic arbor formation started after four days in culture, the molecular events associated with dendritic arborization were induced between DIV 5 and 6. Thus, six days of incubation was chosen for proteomic analysis^12^. Around 800 protein spots were detected in each gel and 300 spots were differentially expressed (Fig. 4a). With the fold change of >1.5, a total of 17 protein spots were chosen for identification by MALDI-TOF-MS and PMF. Of these, sixteen proteins were upregulated and one downregulated (Fig. 4b, also indicated by circles in Fig. 4a). Magnified images of the identified protein spots and the relative spot intensities were illustrated in Fig. 4c,d. The characteristics of the identified proteins are summarized in Table 1.

**Table 1:** List of differentially expressed proteins in primary culture of hippocampal neurons treated with SCH vs vehicle.

Spot no.	Serial no.	Gi-accession no.	Identified protein	Gene symbol	Mascot score	Observed Mr (kD) /pI	Protein expression ratio (SCH/vehicle)	Cellular/Biological function
1406	1	51948476	Cytochrome b-c1 complex subunit 1, mitochondrial precursor	Uqcrfs1	191	53.5/5.57	2.7	Mitochondrial electron transport
1610	2	488838	CaBP1 (Calcium binding protein 1)	Cabp1	194	47.59/4.95	1.7	Inhibits agonist-induced intracellular calcium signaling through regulating L-type calcium channel
1706	3	165971447	MAP1B protein (microtubule-associated protein 1b)	Map1b	250	77.792/8.46	2.3	Neuronal development, microtubule polymerization and stabilization. Autophagy.
2209	4	293342999	Ankyrin domain family member F isoform X1	Potef/Actb	153	42.109/5.31	2.7	Neuronal differentiation and axon guidance
3501	5	40254595	Dihydropyrimidinase-related protein 2	Crmp2	127	62.638/5.95	1419	Axonal growth and guidance
4102	6	149028159	Similar to putative ATP-dependent Clp protease proteolytic (clpP) subunit, mitochondrial precursor	Clpp	100	36.527/7.68	3.1	Proteolysis involved in cellular protein catabolic process. Chaperone function.
5611	7	25742751	Dihydropyrimidinase-related protein 1	Crmp1	261	62.499/6.64	2.2	Cytoskeleton remodeling and axonal guidance
6003	8	14091667	LEK1	cenpf	83	39.504/4.86	1.9	Regulates microtubule function through its interaction with the LIS1 pathway, mainly with NudE
6607	9	25742568	Dihydropyrimidinase-related protein 3	Crmp3	242	62.327/6.04	0.3	Cytoskeleton remodeling and axonal guidance
6705	10	84781723	Heat shock protein 75 kDa, mitochondrial precursor	Trap1	139	80.639/6.56	1182	Stress responses, intracellular trafficking, and protein folding
6709	11	170785225	Chain A, revised structure of the Munc18a-Syntaxin1 complex	Stx1a	159	69.323/6.76	1.7	Synaptic vesicle docking
7002	12	71121745	Hnrph1 protein (heterogeneous nuclear ribonucleoprotein H1)	Hnrnph1	141	20.795/5.20	2.2	Regulation of RNA splicing. Involved in neurogenesis, differentiation and synaptogenesis
7102	13	8394076	Proteasome subunit alpha type-6	Psma6	161	27.838/6.34	2.1	Proteolysis in a non-lysosomal pathway
7305	14	6978491	Aldose reductase	Akr1b7	115	36.230/6.26	2.6	Xenobiotic metabolism
8005	15	4504447	Heterogeneous nuclear ribonucleoproteins A2/B1 isoform A2	Hnrnpa2b1	107	36.041/8.67	5.1	Regulation of RNA splicing. Involved in neurogenesis, differentiation and synaptogenesis
8604	16	12711692	Dihydropyrimidinase-related protein 5	Crmp5	86	62.071/6.60	3.7	Remodeling of the cytoskeleton
9501	17	189181716	Succinyl-CoA:3-ketoacid coenzyme A transferase 1, mitochondrial precursor	Oxct1	141	56.624/8.70	3.6	Extrahepatic ketone body catabolism

### Validation of proteomics data by immunocytochemical analysis

We then performed immunocytochemistry of hnrnpa2b1 and Map1b to validate their proteomic profile. Both of these proteins were upregulated in proteomic analysis and known to be involved in neurite outgrowth and maturation^30–32^. The expressions of Hnrnpa2b1and Map1b, as calculated by the ratios of the fluorescence intensities of Hnrnpa2b1 to α-tubulin and Map1b to α-tubulin, were significantly higher (*p* < 0.01) in the SCH-treated neurons compared to vehicle-treated neurons (Supplementary Fig. S2), validating their upregulation in proteomic profile.

### Bioinformatics analysis of differential protein profiles

**Figure 5:**
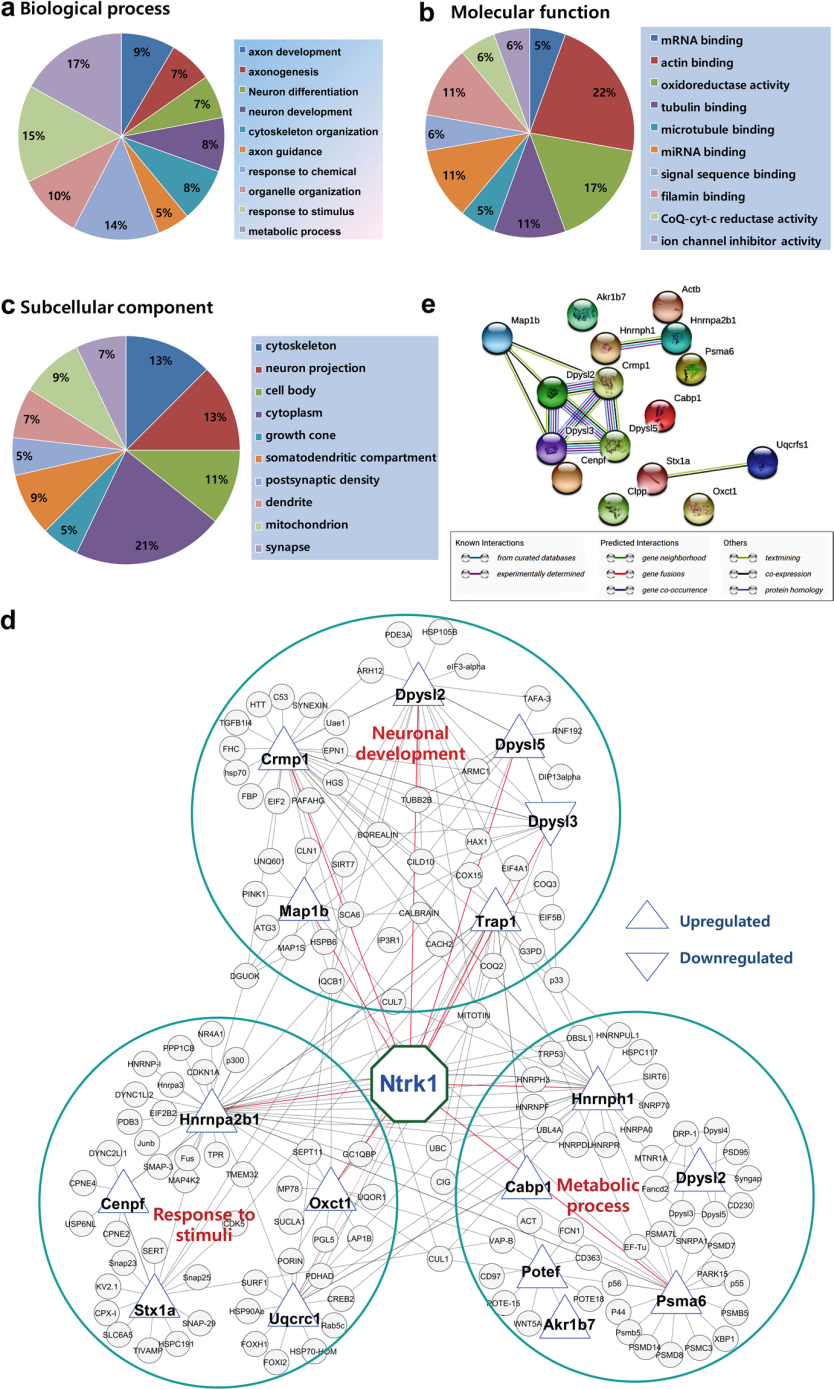
Bioinformatics analysis of the differentially expressed proteins. Functional enrichment of Gene Ontology (GO) by DAVID annotation tool (version 6.8): top 10 GO terms for biological process (**a**), molecular function (**b**) and subcellular component (**c**) were displayed. Interaction networks were visualized by Cytoscape software (version 3.7.1) (**d**). Up and down triangles indicate up- and down-regulated proteins, respectively. Large grouping circles indicate three enriched protein−protein interaction clusters. Here interaction of TrkA (Ntrk1) with the majority of the differentially regulated proteins indicates that SCH might function through TrkA signaling pathway. Protein-protein interaction network analysis by STRING (version 11.0) (**e**).

To characterize differentially expressed proteins, the Gene Ontology (GO) terms including biological process (BP), molecular function (MF) and subcellular component (SC) were analyzed. The highly enriched BPs are those associated with neuronal development and maturation. Moreover, several other biological processes that are enriched during early postnatal neuronal development include metabolic process and stress response (Fig. 5a). The most over-represented MFs are correlated with cytoskeleton formation, particularly microtubule assembly and stabilization (Fig. 5b).

### Protein-protein interaction (PPI) network analysis of differentially expressed proteins

To establish functional links among the differentially expressed proteins and their related biological processes, the PPI network was constructed using Cytoscape. As shown in Fig. 5d, the connection of altered proteins with three major biological processes, including neuronal development, metabolic process and response to stimuli was highly established. Moreover, STRING tool revealed that 2/3^rd^ of the altered proteins were also connected indicating their involvement in multiple biological processes (Fig. 5e). The most striking information obtained from bioinformatics analysis is the appearance of TrkA, a transmembrane growth factor receptor, connecting most of the upregulated proteins in the PPI network (Fig. 5d), which led us to postulate that the neurite outgrowth promoting activity of SCH might be TrkA-dependent.

### Neurite outgrowth activity of SCH is TrkA-dependent

**Figure 6:**
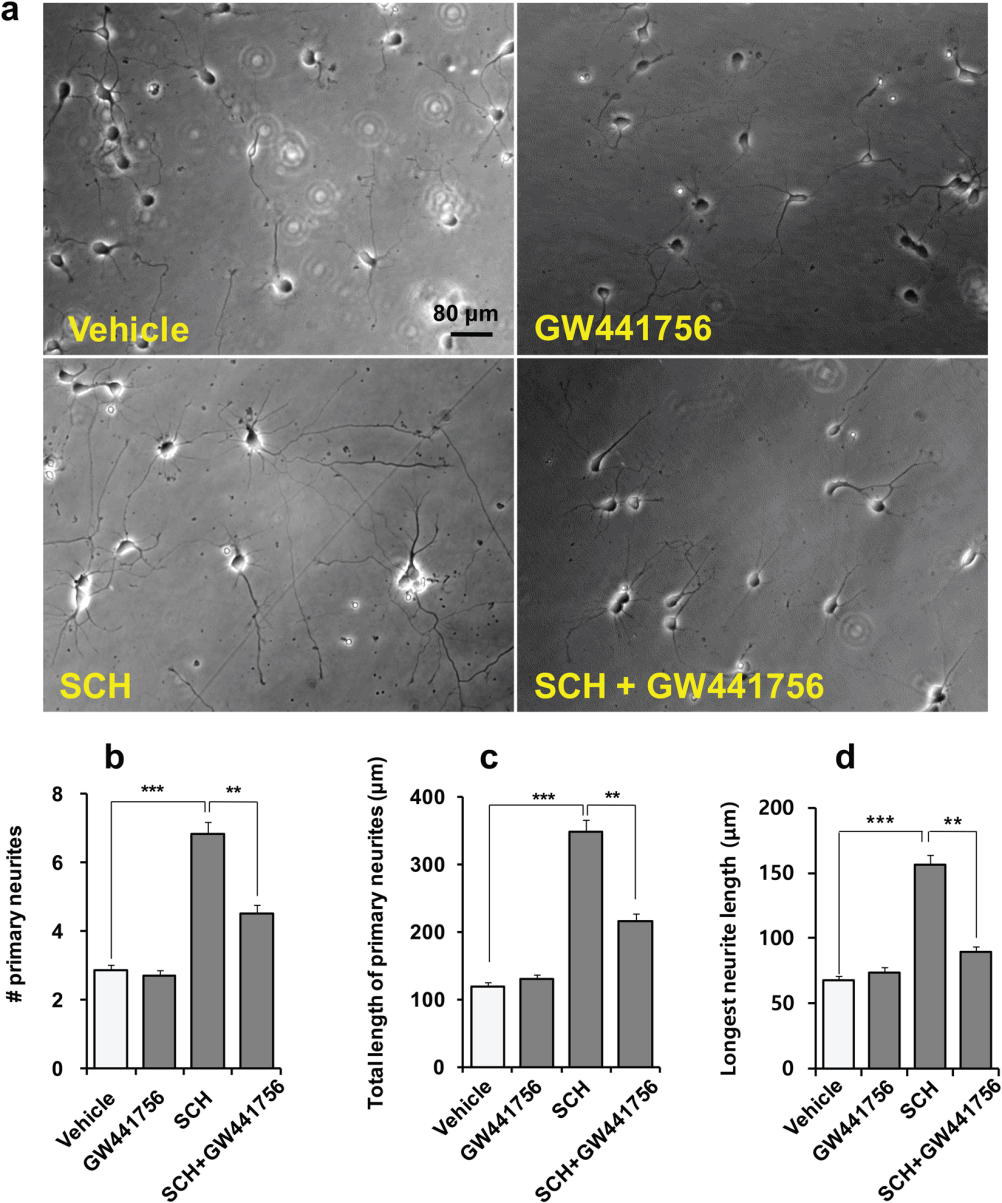
Neurite outgrowth activity of SCH is TrkA-dependent. Hippocampal neurons were grown on the same culture conditions, as indicated in Fig. 1 in the presence of vehicle, GW441756 (TrkA inhibitor, 1 μM), SCH (30 μM) or SCH + GW441756 for two days. Representative phase-contrast photomicrographs from each treatment (**a**). Scale bar, 80 μm, applies to all images. Morphometric analysis for the number (**b**), and the total length of primary neurites (**c**), and longest neurite length (**d**) demonstrates that GW441756 caused a reduction in the activity of SCH on neurite extension. Statistical significance compared to vehicle: **p < 0.01 and ***p < 0.001 (ANOVA). Data points represent the mean ± SEM (n ≥ 20 individual neurons).

Next, we tested whether SCH exerted its neurotrophic effects through this canonical signaling pathway. Treatment of neuronal culture with a specific TrkA inhibitor, GW441756, significantly reversed the stimulatory activity of SCH on neurite extension, showing that SCH-mediated neurite outgrowth involves TrkA signaling pathway (Fig. 6).

### SCH interacts with the NGF-binding domain of TrkA through Phe327 and Asn355

**Figure 7:**
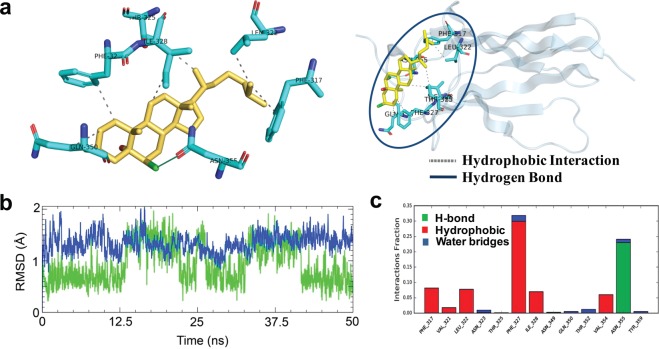
Binding and interaction pattern of SCH at the fifth subdomain in the extracellular domain of TrkA receptor. Three-dimensional bound conformation of SCH in the TrkA, where it established direct interactions through hydrogen and hydrophobic bonds (**a**). Dotted lines represent the type of interactions between binding site residues and ligand. The results of MD simulation inferring as the RMSDs of protein and ligand that describe the overall conformational stability during 50 ns simulation (**b**). SCH mediated interactions to the binding site residues in TrkA receptor, categorized by hydrogen, hydrophobic and water bridge, respectively (**c**). Here each bar represents the percent of bond occupancy as a fraction.

Several earlier reports highlighted the critical contributions of the fifth subdomain (extracellular part) at the extracellular part of TrkA to NGF binding in initiating NGF mediated TrkA activation^33–36^. Furthermore, previous NMR studies also revealed that the fifth subdomain contains small molecule binding site^37^, where agonist can bind and modulate TrkA activity. Thence, molecular modeling studies were further incorporated to get insight into the interaction patterns and efficiency of SCH to the TrkA’s fifth subdomain. Interestingly, molecular docking simulation showed that SCH interacted with the subdomain by forming hydrogen bonding with Asn355 residue through the chlorine group at the position of C3 and C6. Furthermore, the domain serves a hydrophobic cavity. As a result, the compound also showed hydrophobic interactions with Leu322, Thr325, Phe317, Phe327, Ile328 and Gln350 residues, respectively, (Fig. 7a) where Thr325 and Phe327 directly involved to the contact with NGF^33^. The compound showed binding affinity and MM/GBVI binding energy of −6 kcal/mol and −30 kcal/mol, respectively^38^. In order to validate this finding, additional molecular dynamics simulation was conducted for 50 ns and RMSD values for both protein and ligand were initially calculated for system stability. As shown in Fig. 7b, the compound, SCH formed stable interaction to the binding site of TrkA during the simulation. Although the compound was flexible during the simulation, no significant change in protein conformation was observed. Moreover, the compound maintained the highest polar interaction with Asn355 residue in the simulation, which was mediated through halogen substitution at C3 and C6, as revealed by the molecular docking analysis. Besides, Phe327 showed maximum hydrophobic interaction with SCH (Fig. 7c), suggested that the compound binds to the NGF binding domain of TrkA and could function as NGF mimetic.

Next, we designed TrkA truncate through computational mutagenesis, which allowed us to prove the interaction of SCH with the wild type TrkA. We employed alanine (Ala) scanning mutagenesis, one of the most powerful *in silico* mutagenesis, which replaces an amino acid residue to Ala^39^. Since Phe317, Leu322, Phe327, Ile328, Val354, and Asn355 residues formed maximum non-bonded interactions with the ligand SCH as revealed by the protein-ligand interactions profile followed by 50 ns molecular dynamics simulation, these residues were replaced with Ala and ligand binding energy was calculated by the MM-GBSA approach in order to elucidate the variation in the binding affinity. The MM-GBSA calculation revealed that mutation of all residues to Ala decreased the binding energy of the ligand (Supplementary Table S1). The highest reduction was observed for the F327A and N355A residues, which indicates that Phe327 and Asn355 play a crucial role in ligand binding. Furthermore, these residues had shown maximum interactions in molecular dynamics simulation. Together, these findings suggest that SCH interacts with the wild type TrkA.

## Discussion

We demonstrate that SCH promoted the differentiation and maturation of hippocampal neurons in primary culture. The competence of neurons against the physiological loss was also elaborated by SCH application. The promotion by SCH of both neurite outgrowth and cell survival indicates that SCH has both the neurotrophic and neuroprotective potentials.

Neurons are highly polarized cells, whose polarity is achieved through a stereotypic progression of events beginning with neurite sprouting (stage I & II), axonal differentiation (stage III), dendrite arborization (Stage IV) and synaptic formation (stage V)^29^. Here, we observed that SCH contributed to every stage of neuronal development under investigation.

Neurodegenerative disorders, as well as acute brain injury, are accompanied by extensive damage to the neuronal network. Evidence support that neurotrophin mimetics can restore partially damaged neuronal network through dendritic remodeling and axonal regeneration^40^. Moreover, in the experimental stroke model, neurotrophin mimetics are shown to have neuroprotective roles^3,6^. In the present study, neurons treated with SCH showed well-developed axon and dendrites with higher branching frequency forming a more extensive neuronal network. Thus, SCH could offer therapeutic means through the reconstruction of neuronal circuitry, often compromised in neurodegenerative diseases and acute brain injury.

Neuronal differentiation and development during the early postnatal period involve diverse signaling pathways and interplay of multiple proteins. Using a proteomic approach, we demonstrated a differential expression pattern of 17 proteins in SCH-treated primary hippocampal neurons. Gene ontology-based bioinformatics analysis of these proteins indicated that neuronal morphology-related biological processes such as axon development, axonogenesis, axon guidance, neuronal differentiation, and neuronal development were the highly over-represented cellular process. Among the altered proteins, Potef, Crmp1, Crmp2, Crmp5, Map1b, Hnrnpa2b1, and Hnrnph1 have a direct correlation with these biological processes. Several of those are associated with microtubule formation and thus help maintain neuronal cytoskeleton. For instance, Crmp2 (also known as Dpsyl2) is an axon-specific protein that promotes assembly of tubulin dimer into microtubule polymers. Overexpression of Crmp2 enhances axonal growth and may even cause a transformation of already established dendrites into aberrant axons^41–43^. Multiple axons were also observed in SCH-treated neurons, which might be explained by the highly expressed Crmp2 in proteomic profile by SCH treatment. Similarly, Map1b, as an important microtubule-associated protein (MAP), maintains a dynamic equilibrium between cytoskeletal components, and regulate the stability and interaction of microtubules and actin to modulate axonal growth and neuronal connectivity^30,31^. Map1b predominantly expressed in the growth cone than in elongating axons^44,45^ and its knockout compromises axonal development, which together indicate the critical role of Map1b in axonogenesis and elongation^46^. Furthermore, Map1b overexpression leads to an increase in microtubule elongation rates, thus enhances the rates of microtubule assembly as well as axonal elongation in developing neurons^47^. Hou and colleagues reported that the basal level of Map1b, a fragile X mental retardation protein (FMRP) binding mRNAs in hippocampal area CA1 of *Fmr1* knockout mice is elevated compared to that of wild-type mice^48^. However, the outcome of this overexpression of Map1b in the physiological condition *in vivo* may be beneficial but need to be investigated further. Notably, both Map1b and Crmp2 are involved in the signaling pathways that govern axon specification during neuronal polarization. Therefore, faster axonal sprouting in early stage followed by extensive axonal maturation by SCH treatment might be due to the upregulation of Map1b and Crmp2.

Other upregulated proteins by SCH treatment under Crmp family include Crmp1 (also known as Dpsyl1) and Crmp5 (also known as Dpsyl5) which are downstream mediators of semaphorin3A signaling, an extracellular signal transduction pathway that promotes axonal and dendritic sprouting and guidance^49^. Unlike Crmp2, Crmp1 and Crmp5 are involved in both dendrite and axonal maturation^50^. Crmp1 takes part in dendritic development as Sema3A is implicated in dendritic spine maturation^51^. Thus, Crmp1 and Crmp5 might play a crucial role in SCH-mediated neuronal differentiation and maturation. Although Crmp3 (also known as Dpsyl3) is involved in cytoskeleton development, it was downregulated in our study. Crmp3 might be involved in the differential regulation that is essential for proper neuronal development. Like Crmp family proteins, Lek1 (also known as Cenpf) is another upregulated MAP in SCH-treated culture that regulates microtubule function through its interaction with LIS1 pathway, mainly with NudE^52^. Moreover, ankyrin F isoform X1 (also known as potef or Actb), which is critical for neuronal differentiation and axon guidance^53^ might be involved in SCH-mediated axonal sprouting and maturation.

RNA-binding proteins (RBPs) function as essential mediators in the protein expression in neurons through their roles in the post-transcriptional modification, i.e., mRNA splicing. With their regulatory role in gene expression, RBPs are known to coordinate the spatiotemporal differentiation of neurons^32^. Their functions span every stage of development from neuronal differentiation to synaptic plasticity. In the present study, the high expression of Hnrnpa2b1 and Hnrnph1 by SCH treatment, thus, indicates an association between RBPs and increased neuritogenesis in SCH-treated hippocampal neurons.

Hippocampal neurons treated with SCH showed upregulation of energy-efficient metabolic enzymes including cytochrome b-c1 complex subunit 1(also known as Cytb-c1 or Uqcrfs1) and succinyl-CoA:3-ketoacid CoA transferase 1, mitochondrial precursor (Oxct1). Uqcrfs1 is a part of the complex III of mitochondrial electron transport chain and involved in oxidative phosphorylation^32^. On the other hand, Oxct1 is a key enzyme for the extrahepatic ketone body catabolism in which it catalyzes the reversible transfer of CoA from succinyl-CoA to acetoacetate. These outcomes are consistent with energy-intensive processes associated with early postnatal neuronal development and survival. However, the upregulation of Oxct1 in SCH-treated culture could be explained by the fact that neurons were grown with the plating media without further feeding for subsequent 6 days, which left the culture relatively energy depleted especially in the SCH-treated culture where neurons grew faster than that of vehicle counterpart as early postnatal neuronal development is a high energy-demanding process. So, in that case, neurons were furnished with an excess of the ketone body catabolism-related enzymes as a compensatory response to energy insufficiency.

Several proteins related to stimulus-response were also upregulated in SCH-treated neurons. For instance, heat shock protein 75 (Hsp75) under Hsp70 chaperone family is predominantly expressed in mitochondria in response to stress. Hsp75 is not heat-inducible, instead responds to other forms of stress, including energy deficiency, and certain drugs^54^. HSPs help the folding of misfolded proteins by preventing their aggregation. Thus, SCH-mediated upregulation of Hsp75 might play a significant role in regulating proper protein folding during high protein turnover in neuronal differentiation.

Neuronal autophagy plays a vital role in axonal homeostasis during development^55^. Besides of its role in microtubule stabilization, Map1b in association with LC3 participates in autophagosome trafficking in the axon^55^. Wang and colleagues observed that the overexpression of Map1b significantly declines the number of LC3-associated autophagosomes in non-neuronal cells, seemingly through the Map1b-LC3 interaction, which participates in the remodeling of axonal structures^56^. On the other hand, proteasome subunit alpha type-6 **(**Psma6) is one of the essential subunits of 20S proteasome complex which enzymatically processes abnormal proteins in a non-lysosomal pathway^57,58^. It has been reported that neuronal differentiation in culture is accompanied by increased proteasome activity^59^. Both of these proteins related to protein degradation system were upregulated in SCH-treated neurons. However, these stress-related proteins were overexpressed, at least in part, to cope with any adverse consequence associated with relative energy depletion, for instance, experienced by SCH-treated culture as mentioned earlier.

Along with these stress-related proteins, neurons treated with SCH also exhibited upregulation of some other proteins that are involved in the complex regulatory system. For instance, aldo-keto reductase family 1, member 7 (Akr1b7) has been suggested to contribute to the detoxification of lipid peroxidation by-products^60^, and thus its upregulation by SCH treatment might help to neutralize the excess lipid peroxidation by-products originated due to the higher metabolic activity in culture. Another upregulated protein is Cabp1, which inhibits agonist-evoked IP3-mediated intracellular calcium signaling^61^. This inhibition might be crucial for neuronal differentiation and growth, as well as survival.

Stx1a is critically implicated in the maintenance of developing and mature neurons and in the docking of synaptic vesicles at presynaptic zones^62,63^. We observed SCH-mediated upregulation of Stx1a even in this early phase of neuronal development, indicating a provoking role of SCH in the further neuronal maturation.

In the protein-protein interaction network mapped through Cytoscape, it has been observed that tropomyosin receptor kinase A, TrkA connected most of the altered proteins. TrkA is a transmembrane growth factor receptor that is involved in the development of central nervous systems through regulating differentiation, maturation, and survival of neurons. To confirm the involvement of TrkA in SCH-mediated neurite outgrowth, we applied a TrkA-specific inhibitor, GW441756 in hippocampal culture. The results demonstrate that the effect of SCH was significantly suppressed by GW441756, an effect which confirmed that enhanced neurite outgrowth was due to TrkA activation by SCH.

Once activated, TrkA, in turn, activates a number of protein kinases including protein kinase A (PKA), mitogen-activated protein kinase (MAPK), calmodulin-dependent protein kinase (CaMK), and other kinases^64^, which phosphorylate cAMP-response element binding protein (CREB). As a transcription factor, pCREB binds to the cAMP response element (CRE) of the promoters of its target genes and transcription is initiated^65^. Interestingly, most of the differentially expressed proteins in our study are the targets of CREB at the transcription level^66^. A line of evidence suggests a close correlation between the CREB activity and the biological processes, including differentiation, survival, long-term synaptic potentiation, and neuronal plasticity^64,67,68^. Together this information further supports that SCH might exert its neurotrophic activity through TrkA signaling pathway. In addition, our *in silico* data indicate that SCH interacts with the NGF-binding domain of TrkA. Scarpi and colleagues identified NGF-mimetic small molecule agonist of TrkA, proposing the binding groove at fifth subdomain^69^. In later studies, Pediaditakis and team also recognized a C17-spiroepoxy steroid derivative as NGF mimetic acting on the same binding site and concluded as the role of 5-androstene skeleton of steroid compound to bind and activating of TrkA receptors^70^. Several other agonists, including amitriptyline and LM22A, are also reported to activate Trk through interacting extracellular domain^17,18,21^. Moreover, computational mutagenesis with Ala substitution in the ligand binding residues indicates that the highest reduction in the binding energy of the SCH was observed in F327A and N355A residues, further suggesting the involvement of Phe327 and Asn355 in the SCH binding to wild type TrkA. Together with these reports, the present findings indicate that SCH promoted neuronal development in a TrkA-dependent manner.

## Conclusion

**Figure 8:**
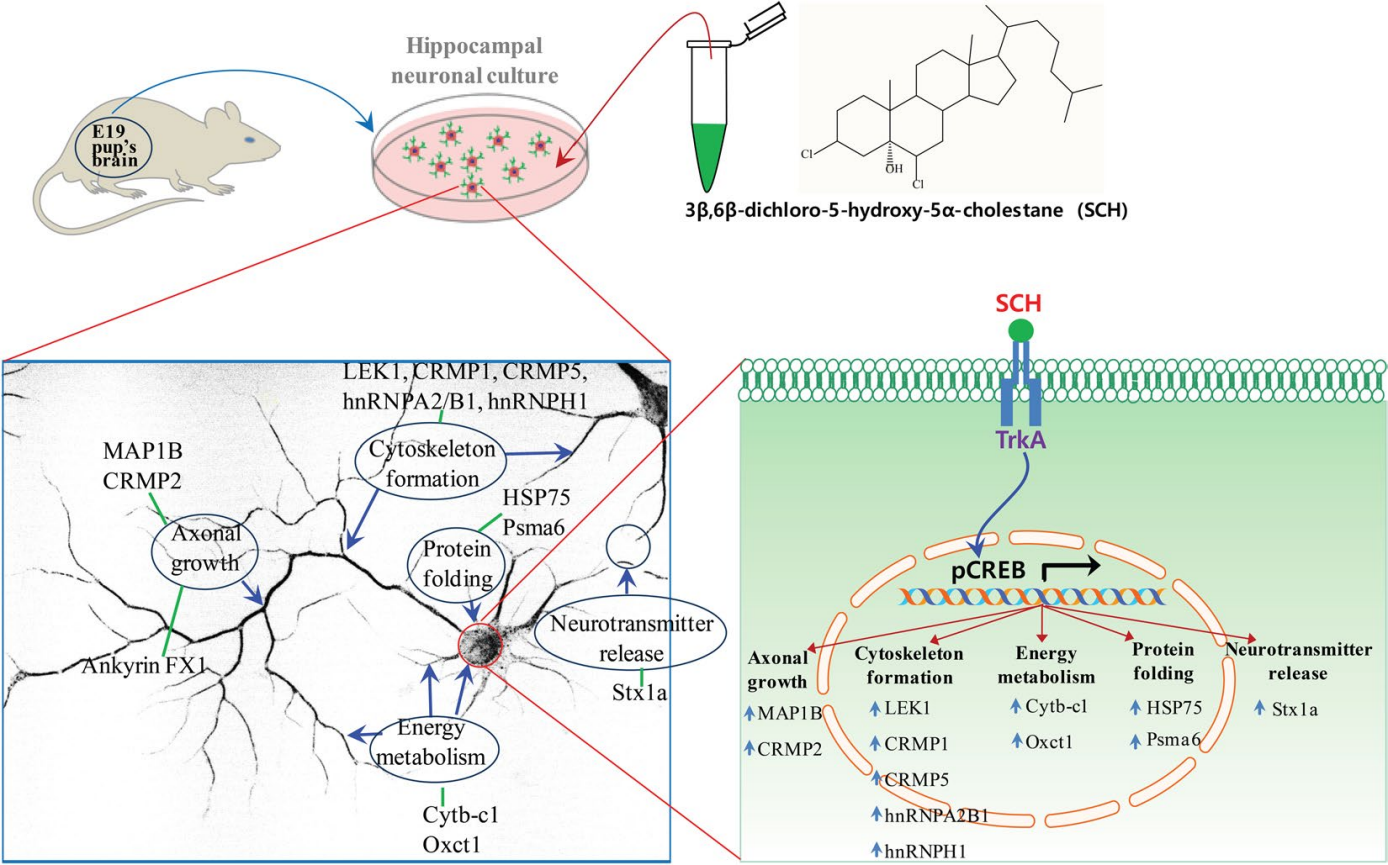
Illustration on the possible underlying pharmacological mechanism of neurotrophic activity of SCH in cultured hippocampal neurons. CREB activated upon stimulation of TrkA signaling pathway by SCH transcribes most of the differentially expressed proteins in the present proteomic study, indicating that SCH might show neurotrophic activity through this canonical pathway.

This study demonstrates that SCH enhanced the development of primary hippocampal neurons through modulating early neuronal differentiation, dendritic arborization and axonal maturation. As illustrated in Fig. 8, these neuromodulatory effects of SCH might be associated with the neuronal polarity (Map1b, Crmp1, Crmp2, Crmp5, Hnrnpa2b1 and Hnrnph1), energy metabolism (Cytb-c1 and Oxct1), protein folding (Hsp75 and Psma6) and neuronal connectivity (Stx1a). We also confirmed that TrkA signaling pathway is critical for SCH-mediated neurotrophic activity. Thus, our findings on the neurotrophic role of SCH in hippocampal neurons might have clinical significance for the treatment of various neurodegenerative disorders and acute brain injury.

## Materials and Methods

### Chemicals and reagents

All chemicals and reagents were procured from Invitrogen (Carlsbad, CA, USA) unless stated otherwise. The 3β, 6β-dichloro-5-hydroxy-5α-cholestane was synthesized using protocol cited in the literature^71^ and characterized by various spectroscopic techniques including a single X-ray crystallography^72^. An aliquot of 17 mM stock solution of SCH was prepared in DMSO and stored at −20 °C for further experiments.

### Primary neuronal culture and compound treatment

All procedures followed the Principles of Laboratory Animal Care (NIH, Washington, DC, USA) and were approved by the Institution Animal Care and Use Committee of Dongguk University (approval certificate number IACUC-1909-5). Time-pregnant rats (Sprague-Dawley) were ordered on the 13^th^ day of pregnancy and housed in controlled temperature with a light/dark cycle of 12/12 h and with access to food and water *ad libitum*. After six days (19^th^ day of pregnancy), the pregnant rat was euthanized with isofluorane and the fetuses were collected. The fetal hippocampi were then dissected from the brain and dissociated neuronal cultures were prepared as previously described^8,73^. Briefly, the dissected hippocampi were collected in Hank’s balanced salt solution (HBSS), and the tissues were dissociated by trypsinization (0.25% trypsin in HBSS) for 12 min at 37 °C and trituration with fire-polished graded Pasteur pipettes. The dissociated cells were seeded onto poly-DL-lysine (Sigma-Aldrich, St. Louis, MO) coated Ø12-mm glass coverslips in 24-well culture plates at a density of 1.0 × 10^4^ cells/cm^2^ for the morphological study or 3.0 × 10^4^ cells/cm^2^ for viability assay and proteomic data validation or in 6-well culture plates at 1.0 × 10^6^ cells/cm^2^ for proteomic analysis. Cells were seeded in preincubated plating media (serum-free neurobasal media supplemented with B27, glutamate and β-mercaptoethanol) and incubated at 37 °C under 5% CO_2_ and 95% air. In case of protein isolation for proteomics, plating media was exchanged with maintenance media (serum-free neurobasal media supplemented with B27) after 6 hrs of cell seeding. Compound or vehicle (DMSO, final concentration <0.5%) were added to the media before cell seeding. A basal control (media only) and a vehicle control (media with DMSO) cultures were always compared with those treated with the test compound.

### Fluorescence labeling of neurons with DiO

Neurons at three days *in vitro* (DIV3) were live-stained with Vybrant DiO (Molecular Probes, Invitrogen) following the manufacturer’s instructions. DiO is a lipophilic dye that binds to the plasma membrane of cells, and thus, was used to stain the entire neuron for morphological analysis.

### Analysis of neuronal viability

Neuronal cultures were incubated with either vehicle or SCH at a varying concentration ranging from 30 to 120 μM. At DIV7, neuronal viability was determined by trypan blue exclusion assay following the procedure as described previously^11^. A comprehensive protocol is, however, included in the Supplementary Methods section.

### Evaluation of cytotoxicity

The extent of cellular injury was determined through CytoTox96 nonradioactive assay (Promega, Madison, WI) by measuring the activity of LDH released in the media of neuronal culture maintained with the same culture condition as in viability analysis. LDH activity (equivalent to cytotoxicity) was expressed in percentage of the ratio of experimental LDH release with maximum LDH release. Data are normalized to the quantity of LDH released from vehicle-treated cells (100%).

### Immunocytochemistry

At the indicated time, neurons on coverslips were rinsed briefly with D-PBS and fixed by a sequential paraformaldehyde/methanol fixation procedure^69^. The following antibodies were used for immunostaining: primary antibodies to tubulin α-subunit (mouse monoclonal 12G10, 1:1000 dilution; Developmental Studies Hybridoma Bank, University of Iowa, IO), ankyrin G (rabbit polyclonal H-215, 1:50 dilution; Santa Cruz Biotechnology Inc., CA), MAP2 (mouse monoclonal clone HM-2; 1:500; Sigma, MO), Tau (rabbit polyclonal LF-PA0172; 1:500; Abfrontier, Seoul, Korea), Hnrnpa2b1 (Goat polyclonal, 1:500; Santa Cruz Biotechnology Inc., CA), Map1b (rabbit polyclonal; 1:500; Cell Signaling Technology, Danvers, MA), and secondary antibodies (Alexa Fluor 488-conjugated goat anti-mouse IgG [1:1,000], Alexa Fluor 568-conjugated goat anti-rabbit IgG [1:1,000], Alexa Fluor 568-conjugated rabbit anti-goat IgG [1:1,000], Molecular Probes, OR). Neurons were then incubated with primary antibodies followed by secondary antibodies and mounted on slides as previously described^74^.

### Microscopic image acquisition, analysis and quantification

A detailed procedure for acquisition, analysis and quantification of the microscopic image is described in the Supplementary Methods section.

### Proteomic analysis of the SCH-treated neurons

A 2DE-based proteomic study was performed to investigate the changes in cellular proteome to elucidate the underlying pharmacological mechanism of neurotrophic activity of SCH in hippocampal neurons, as described previously^12^. A detailed protocol is described in the Supplementary Methods section.

### Bioinformatics analysis of differentially expressed proteins

Gene Ontology (GO) terms enrichment analysis including biological process, molecular function, and subcellular component was carried out using the Database for Annotation, Visualization and Integrated Discovery (DAVID) version 6.8^75^ and Enrichr^76^. GO terms with a *p*-value of < 0.05 cut off were considered significant. Networks enriched with significantly altered proteins were visualized by Cytoscape v3.7.1^77^. The analysis of protein-protein interaction network of differentially expressed proteins was carried out using STRING version 11.0.

### Virtual screening and molecular dynamics simulation

An *in silico* analysis was performed to elucidate whether SCH interacts with TrkA. The detailed procedure is described in the Supplementary Methods section.

### *In silico* mutagenesis studies

Computational mutagenesis by alanine substitution was carried out to elucidate the contribution of active site residues to the ligand binding. The detailed procedure is described in the Supplementary Methods section.

### Statistical analysis

Data are expressed as the mean ± SEM with at least three independent experiments unless stated otherwise. Statistical comparisons were made by Student’s *t-*test, and one-way analysis of variance (ANOVA) with *post hoc* Duncan multiple comparisons (SPSS software, version 16.0). Predetermined *p* values ≤ 0.05 were considered statistically significant.

## Supplementary information

Supplementary information

## Data Availability

All data generated or analyzed during this study are included in this published article (and its Supplementary Information Files).
