# Supplementary material for: 3β, 6β-dichloro-5-hydroxy-5α-cholestane facilitates neuronal development through modulating TrkA signaling regulated proteins in primary hippocampal neuron
Source: Sci Rep. 2019 Dec 12;9:18919. doi: 10.1038/s41598-019-55364-8 (PMC6908615; doi:10.1038/s41598-019-55364-8)
Supplement: Supplementary file 1 — Supplementary information [file 41598_2019_55364_MOESM1_ESM.docx]

**Supplementary file-1**

**3β, 6β-dichloro-5-hydroxy-5α-cholestane facilitates neuronal development through modulating TrkA signaling regulated proteins in primary hippocampal neuron**

Md. Abdul Hannan^1,2^, Md. Nazmul Haque^1,4^, Raju Dash^1^, Mahboob Alam^3^ & Il Soo Moon^1*^

^1^Department of Anatomy, Dongguk University College of Medicine, Gyeongju 38066, Republic of Korea

^2^Department of Biochemistry and Molecular Biology, Bangladesh Agricultural University, Mymensingh-2202, Bangladesh

^3^Division of Chemistry and Biotechnology, Dongguk University, Gyeongju 780-714, Republic of Korea

^4^Dept. of Fisheries Biology and Genetics, Patuakhali Science and Technology University, Patuakhali-8602, Bangladesh

****Correspondence to*:**

Il Soo Moon

Department of Anatomy

Dongguk University College of Medicine,

Gyeongju 38066, Korea

Phone: +82-54-770-2414

Fax: +82-54-770-2447

Email: [moonis@dongguk.ac.kr](mailto:moonis@dongguk.ac.kr)

**Supplementary Methods**

**Neuronal viability using trypan blue exclusion assay**

The cultures were stained with 0.4% trypan blue for 10 min at RT. The cultures were then washed with Dulbecco’s phosphate buffered saline (D-PBS, Invitrogen), fixed with 4% paraformaldehyde, and quantified under a light microscope. Dead neurons are compromised to membrane permeability, and thus, uptake dye and look dark-blue in color. Live neurons have intact membrane integrity, and therefore, exclude dye. The viability was calculated as the percentage of the ratio of the number of unstained cells (live neurons) to the total number of cells counted (live plus dead neurons) ^1^. In each experiment, cells on three coverslips, 300-400 cells per coverslip, were counted randomly.

**Microscopic image acquisition, analysis and quantification**

A Leica Research Microscope DM IRE2 equipped with I3 S, N2.1S, and Y5 filter systems (Leica Microsystems AG, Wetzlar, Germany) was used for phase-contrast and epifluorescence microscopy. Images (1,388 x 1,039 pixels) were acquired with a high-resolution CoolSNAP^TM^ CCD camera (Photometrics Inc., Munchen, Germany) under the control of a computer using Leica FW4000 software. The digital images were processed using Adobe Photoshop 7.0 software.

Morphometric analyses and quantification were performed with an Image J (version 1.49) software with the simple neurite tracer plug-in (National Institute of Health, Bethesda, MD) and Sholl plug-in (http://biology.ucsd.edu/labs/ghosh/software). Morphometric parameters such as the number of primary dendrites (neurites that originated directly from the soma), the total length of primary dendrites (sum of the length of primary neurites), and axonal length were measured. Axonal and dendritic branching orders were also analyzed. Primary branches are those that originated from primary neurites; secondary and tertiary branches are those that originated from the primary and secondary branches, respectively. To evaluate the degree of arborization of the axonal and dendritic tree, we used Sholl’s analysis^2^. The axonal or dendritic intersection is the point where an axon, dendrite, or their branch intersects the given concentric circle. The numbers of branching points between two successive concentric circles, i.e., within each successive 10-μm radial circle, were counted. Neurons (a minimum of 30 cells) that were not intermingled with the processes of adjacent neurons were selected for analysis. The neuronal cell populations at different developmental stages^3^were counted on captured images. Fluorescent intensities were measured along the length of the neurites. Intensity ratios for Hnrnpa2b1/α-tubulin and Map1b/α-tubulin were then calculated. In our study, neurons of media only and vehicle (DMSO, <1.0%) controls exhibited very similar patterns of growth. Therefore, we always compared extract-treated cultures with vehicle control during morphometric analysis.

**Proteomic analysis of the SCH-treated neurons**

**Chemicals and reagents**

Urea, Thiourea, CHAPS, DTT, Benzamidine, Acrylamide, Iodoacetamide, Bis-acrylamide, SDS, acetonitrile, trifluoroacetic acid, α-cyano-4-hydroxycinnamic acid were purchased from Sigma-Aldrich (Electrophoresis grade, ACS reagents, Ultrapure). Pharmalyte (pH 3.5-10) was from Amersham Biosciences. Modified porcine trypsin(sequencing grade) was from Promega.

**Extraction of protein sample**

At DIV6, protein was harvested from cultured neurons using the Nuclei EZ Prep Nuclei Isolation Kit (Sigma-Aldrich). Protein concentration was determined using the Bradford method^4^.

**Two dimensional gel electrophoresis (2-DE) and image analysis**

2D-PAGE was performed on whole-cell lysate protein fractions. Immobilized pH gradient (IPG) dry strips (4–10 NL IPG, 24 cm; Genomine, Inc., Pohang, Korea) were equilibrated for 12–16 h in a 7M urea, 2M thiourea solution containing 2% 3-[(3-cholamidopropyl) dimethylammonio]-1-propanesulfonate (CHAPS), 1% dithiothreitol (DTT), and 1% pharmalyte and loaded with 200 μg of samples. Isoelectric focusing (IEF) was performed at 20°C using a Multiphor II electrophoresis unit and EPS 3500 XL power supply (GE Healthcare, Little Chalfont, UK), according to the manufacturer’s instructions. For IEF, the voltage was linearly increased from 150 to 3,500 V over 3 h for sample entry followed by a constant 3,500 V. Complete focusing was achieved at after 96 kVh. Before the second dimension, strips were incubated for 10 min in equilibration buffer (50mMTris-Cl, pH 6.8 containing 6 M urea, 2% SDS, and 30% glycerol), first with 1% DTT and second with 2.5% iodoacetamide. Equilibrated strips were inserted into SDS-PAGE gels (20×24 cm, 10–16%). SDS-PAGE was performed using the Hoefer DALT 2D system (Amersham Biosciences), according to the manufacturer’s instructions. Two-dimensional gels were run at 20°C for 1,700 Vh and then silver stained as described^5^. Quantitative analysis of digitized images was carried out using PDQuest software (version 7.0, Bio-Rad Laboratories, Hercules, CA, USA), according to the manufacturer’s instructions. Spot intensities were normalized versus total valid spot intensities. Protein expressions over or under 1.5-fold versus vehicle control were further analyzed.

**Identification of differentially expressed proteins by MALDI-TOF-MS and PMF**

For protein identification by peptide mass fingerprinting (PMF), protein spots were excised, digested with trypsin, mixed with a cyano-4-hydroxycinnamic acid in 50% acetonitrile containing 0.1%TFA, and subjected to matrix-assisted laser desorption/ionization time-of-flight mass spectrometry (MALDI-TOF-MS) (Microflex LRF20; Bruker Daltonics, Billerica, MA, USA), as described^6^. Spectra were collected using 300 shots per spectrum over them/z range 600–3,000 and calibrated by two-point internal calibration using trypsin autodigestion peaks (m/z 842.5099 and 2211.1046). Peak lists were generated using FlexAnalysis version 3.0. The thresholds used for peak-picking were as follows: 500 for a minimum resolution of monoisotopic mass and 5 for S/N. The search program MASCOT, developed by The Matrix Science (www.matrixscience.com/), was used for protein identification by PMF. The following parameters were used for the database search: trypsin as the cleaving enzyme, a maximum of one missed cleavage, iodoacetamide (Cys) as a complete modification, oxidation (Met) as a partial modification, monoisotopicmasses, and amass tolerance of–0.1Da. PMF protein identification was accepted by probability-based scoring.

**Virtual screening and molecular dynamics simulation**

In order to perform molecular docking analysis, crystal coordinates of TrkA ligand binding domain with nerve growth factor was retrieved (PDB ID: 1WWW) and fixed the structural errors by adding charges and hydrogen. The structure was also refined by previously described methods^7,8^ and minimized by applying OPLS 3 force field to adjust the maximum heavy atom RMSD to 0.30 Å. The structure of SCH was drawn by ChemDraw and the conformation was generated by geometry optimization by Jaguar software using Becke's three-parameter exchange potential^9^ and Lee-Yang-Parr correlation functional (B3LYP) theory^10,11^ with 6-31G* basis set^12^. Molecular docking was performed by AutoDock Vina^13^ plugin in UCSF Chimera^14^. Prior to the run Gasteiger charges^15^ were added to the molecules. The range of grid box for docking was kept in maximum to ensure the blind docking. Default setting was maintained for running docking calculation through the shell script provided by the developer. The best pose of the ligand having the lowest binding affinity was further confirmed^16,17^. The final docked complex was evaluated by the generalized Born/volume integral (GB/VI) implicit solvent method was, using the force field of Amber10:EHT with R-Field solvation^18^. After that, 50 ns molecular dynamics simulation using Desmond v3.0 was carried out to characterize the intermolecular interaction patterns between protein-ligand, where the complex was emerged into the TIP3P solvent model that is extended approximately 10Å in each direction. The counter ions (Na^+^/Cl^-^ions) were added to the system to maintain physiological condition and neutralizing the system. The default MD protocol, consisting of Noose-Hover chain thermostat at 300 K, Martyna-Tobias-Klein barostat at 1.01325 bar, isotropic coupling, Coulombic cutoff at 0.9 nm for NPT ensemble was followed to set up the simulation^9,19^. The initial simulation was performed with the Brownian Dynamics at 10K temperature for 100 ps in order to restrain of solute heavy atoms. In similar approach, small simulation at 12ps was done at 10K temperature in NVT ensemble to restrain solute heavy atoms. After that 12 ps simulation was done at 10K temperature in NVT ensemble with small time steps in order to restrain solute heavy atoms. The next stage was begun followed by similar parameters except NPT. An additional solvate pocket trajectory was done immediately. Subsequently, simulation for 12ps in NPT was performed for restraining solute heavy atoms. In final stages, simulation produced 50 ns of trajectories by following 24ps simulation in NPT with no restraints. The resultant trajectories were then subjected for analysis to measure the protein stability and conformational change by means of RMSD, Root Mean Square Fluctuation (RMSF), SSE (Secondary Structure Elements) by using Simulation Interactions Diagram panel of Schrödinger 2017-1 (LLC, New York, NY, USA).

***In silico* Mutagenesis studies**

Computational mutagenesis by alanine replacement was carried out to elucidate the contribution of active site residues to the ligand binding. For that mutate residues script Schrodinger, LLC, New York, NY, USA was used, which changed the interacted residues to alanine with no local rearrangements ^20,21^ . Subsequently, binding energy calculation by MM-GBSA was performed to analyze the change of binding energy due to mutation. MM-GBSA calculation was carried out by Prime module of Schrodinger-2017 (LLC, New York, NY, USA), where the methods combines OPLSAA molecular mechanics energies (EMM), an SGB solvation model for polar solvation (GSGB), and a non-polar solvation term (GNP) composed of the non-polar solvent accessible surface area and van der Waals interactions ^22-24^. The total free energy of binding:

ΔG_bind_ = G_complex_ – (G_protein_ + G_ligand_), where G = EMM + GSGB + GNP

**Supplementary References**

1 Hannan, M. A. *et al.* Moringa oleifera with promising neuronal survival and neurite outgrowth promoting potentials. *Journal of ethnopharmacology* **152**, 142-150, doi:10.1016/j.jep.2013.12.036 (2014).

2 Sholl, D. A. Dendritic organization in the neurons of the visual and motor cortices of the cat. *Journal of anatomy* **87**, 387-406 (1953).

3 Dotti, C., Sullivan, C. & Banker, G. The establishment of polarity by hippocampal neurons in culture. *The Journal of Neuroscience* **8**, 1454-1468, doi:10.1523/jneurosci.08-04-01454.1988 (1988).

4 Bradford, M. M. A rapid and sensitive method for the quantitation of microgram quantities of protein utilizing the principle of protein-dye binding. *Analytical Biochemistry* **72**, 248-254, doi:<https://doi.org/10.1016/0003-2697(76)90527-3> (1976).

5 Oakley, B. R., Kirsch, D. R. & Morris, N. R. A simplified ultrasensitive silver stain for detecting proteins in polyacrylamide gels. *Analytical Biochemistry* **105**, 361-363, doi:<https://doi.org/10.1016/0003-2697(80)90470-4> (1980).

6 Fernandez, J., Gharahdaghi, F. & Mische, S. M. Routine identification of proteins from sodium dodecyl sulfate-polyacrylamide gel electrophoresis (SDS-PAGE) gels or polyvinyl difluoride membranes using matrix assisted laser desorption/ionization-time of flight-mass spectrometry (MALDI-TOF-MS). *ELECTROPHORESIS* **19**, 1036-1045, doi:10.1002/elps.1150190619 (1998).

7 Dash, R., Junaid, M., Mitra, S., Arifuzzaman, M. & Hosen, S. M. Z. Structure-based identification of potent VEGFR-2 inhibitors from in vivo metabolites of a herbal ingredient. *Journal of molecular modeling* **25**, 98, doi:10.1007/s00894-019-3979-6 (2019).

8 Mitra, S. & Dash, R. Structural dynamics and quantum mechanical aspects of shikonin derivatives as CREBBP bromodomain inhibitors. *Journal of molecular graphics & modelling* **83**, 42-52, doi:10.1016/j.jmgm.2018.04.014 (2018).

9 Basha, S. H., Bethapudi, P. & Majji Rambabu, F. Anti-angiogenesis property by Quercetin compound targeting VEGFR2 elucidated in a computational approach. *European Journal of Biotechnology and Bioscience* **2**, 30-46 (2014).

10 Gill, P. M. W., Johnson, B. G., Pople, J. A. & Frisch, M. J. The performance of the Becke—Lee—Yang—Parr (B—LYP) density functional theory with various basis sets. *Chemical Physics Letters* **197**, 499-505, doi:<https://doi.org/10.1016/0009-2614(92)85807-M> (1992).

11 Stephens, P. J., Devlin, F. J., Chabalowski, C. F. & Frisch, M. J. Ab Initio Calculation of Vibrational Absorption and Circular Dichroism Spectra Using Density Functional Force Fields. *The Journal of Physical Chemistry* **98**, 11623-11627, doi:10.1021/j100096a001 (1994).

12 Raju, D. *et al.* Molecular Insight and Binding Pattern Analysis of Shikonin as a Potential VEGFR-2 Inhibitor. *Current Enzyme Inhibition* **13**, 235-244, doi:<http://dx.doi.org/10.2174/1573408013666161227162452> (2017).

13 Sanner, M. F. Python: a programming language for software integration and development. *Journal of molecular graphics & modelling* **17**, 57-61 (1999).

14 Pettersen, E. F. *et al.* UCSF Chimera--a visualization system for exploratory research and analysis. *Journal of computational chemistry* **25**, 1605-1612, doi:10.1002/jcc.20084 (2004).

15 Dunbrack, R. L., Jr. Rotamer libraries in the 21st century. *Current opinion in structural biology* **12**, 431-440 (2002).

16 Trott, O. & Olson, A. J. AutoDock Vina: improving the speed and accuracy of docking with a new scoring function, efficient optimization, and multithreading. *Journal of computational chemistry* **31**, 455-461, doi:10.1002/jcc.21334 (2010).

17 Hosen, S. M. Z. *et al.* Prospecting and Structural Insight into the Binding of Novel Plant-Derived Molecules of Leea indica as Inhibitors of BACE1. *Current pharmaceutical design* **24**, 3972-3979, doi:10.2174/1381612824666181106111020 (2018).

18 Labute, P. The generalized Born/volume integral implicit solvent model: estimation of the free energy of hydration using London dispersion instead of atomic surface area. *Journal of computational chemistry* **29**, 1693-1698, doi:10.1002/jcc.20933 (2008).

19 Reddy, S. V., Reddy, K. T., Kumari, V. V. & Basha, S. H. Molecular docking and dynamic simulation studies evidenced plausible immunotherapeutic anticancer property by Withaferin A targeting indoleamine 2,3-dioxygenase. *Journal of biomolecular structure & dynamics* **33**, 2695-2709, doi:10.1080/07391102.2015.1004834 (2015).

20 Prime, S. LLC. *New York, NY* (2017).

21 Fujimoto, T., Matsushita, Y., Gouda, H., Yamaotsu, N. & Hirono, S. In silico multi-filter screening approaches for developing novel β-secretase inhibitors. *Bioorganic & medicinal chemistry letters* **18**, 2771-2775 (2008).

22 Vijayakumar, B., Umamaheswari, A., Puratchikody, A. & Velmurugan, D. Selection of an improved HDAC8 inhibitor through structure-based drug design. *Bioinformation* **7**, 134-141 (2011).

23 Dash, R. *et al.* Molecular insight and binding pattern analysis of Shikonin as a potential VEGFR-2 inhibitor. *Current Enzyme Inhibition* **13**, 235-244 (2017).

24 Lyne, P. D., Lamb, M. L. & Saeh, J. C. Accurate prediction of the relative potencies of members of a series of kinase inhibitors using molecular docking and MM-GBSA scoring. *Journal of medicinal chemistry* **49**, 4805-4808 (2006).

**Supplementary Figures**


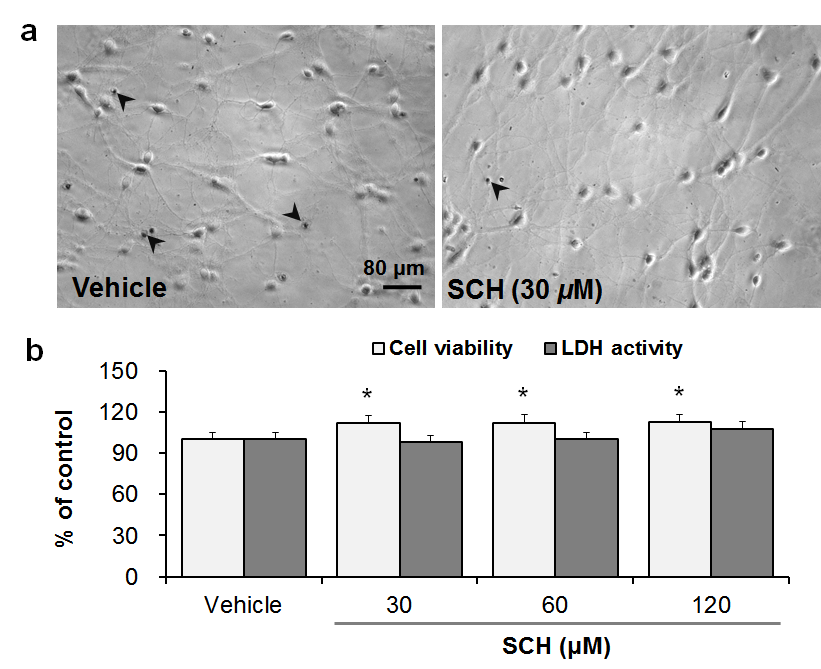


**Figure S1.** Effects of SCH on neuronal viability. Hippocampal neurons were cultured on poly-DL-lysine-coated coverslips with vehicle or SCH for 7 days. Neuronal viability and cytotoxicity were determined in the same culture by trypan blue exclusion and LDH activity assays, respectively. (a) Typical images for trypan blue staining. Arrows indicate dead neurons. Scale bar, 80 μm, applied to both images. (b) The neuronal viability determined as the proportion of the unstained cells (live neurons) to the total number of cells counted (live plus dead neurons); viability of control culture is normalized to 100%. LDH activity equivalent to cytotoxicity is the percentage of the ratio of experimental LDH release with maximum LDH release. Data are normalized to the amount of LDH released from vehicle-treated cells (100%). Bars represent the mean ± SEM (n= 3). Statistical significance compared with vehicle: **p* < 0.05 (ANOVA).

**
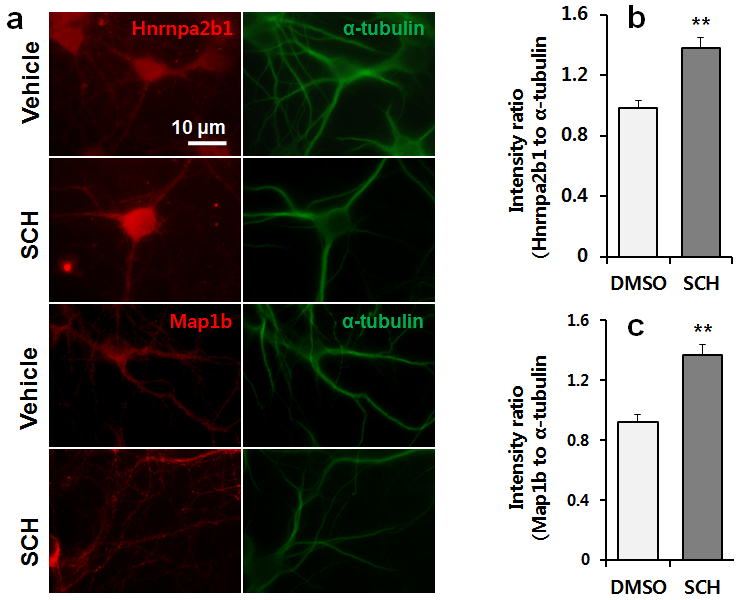
**

**Figure S2.**

Immunocytochemical validation of the expression of some proteomics-identified proteins. Hippocampal neurons were cultured in the presence of vehicle or SCH (30 μM) for 6 days. Neurons were then fixed and double immunostained for Hnrnpa2b1/α-tubulin and Map1b/α-tubulin. (a) Representative fluorescence photomicrographs of Hnrnpa2b1 versus α-tubulin and Map1b versus α-tubulin immunostained hippocampal neurons from vehicle or SCH–treated culture. Scale bar, 10 μm in all images. Fluorescence intensity ratios of Hnrnpa2b1 versus α-tubulin (b) and Map1b versus α-tubulin (c). Statistically significant versus vehicle controls: ***P*<0.01(Student's *t*-test). Bars represent the mean ± SEM (n = 10-12 individual neurons).

**Supplementary Table**

Table S1: Change of binding free energy due to *in silico* alanine scanning mutagenesis

| **Name** | **ΔG_Bind_^a^**  **_(kcal/mol)_** | **ΔG_Bind___Coulomb_^b^** | **ΔG_Bind_Lipo_^c^** | **ΔG_Bind_ __Solv GB_^d^** | **ΔG_Bind___vdW_^e^** |
| --- | --- | --- | --- | --- | --- |
| Wild | -48.35 | -5.23 | -18.77 | 11.53 | -36.03 |
| F317A | -47.21 | -5.08 | -17.5 | 11.15 | -35.9 |
| L322A | -45.88 | -5.25 | -17.48 | 11.67 | -35.04 |
| F327A | -43.2 | -5.24 | -15.87 | 11.33 | -33.48 |
| I328A | -48.76 | -8.42 | -19.11 | 12.02 | -33.6 |
| V354A | -46.36 | -5.41 | -17.71 | 11.68 | -35.11 |
| N355A | -44.00 | -5.15 | -16.75 | 12.03 | -34.21 |

^a^MMGBSA free energy of binding

^b^Contribution to the MMGBSA free energy of binding from the Coulomb energy

^c^Contribution to the MMGBSA free energy of binding from lipophillic binding

^d^Contribution to the MMGBSA free energy of binding from the generalized Born electrostatic solvation energy.

^e^Contribution to the MMGBSA free energy of binding from the van der Waals energy
